# Effects of Dental Rehabilitation under General Anesthesia on Children’s Oral-Health-Related Quality of Life: Saudi Arabian Parents’ Perspectives

**DOI:** 10.3390/dj3010001

**Published:** 2014-12-23

**Authors:** Ziad D. Baghdadi, Nazeem Muhajarine

**Affiliations:** 1 Department of Preventive Dentistry, Riyadh Colleges of Dentistry and Pharmacy, P.O. Box 67126, Riyadh 11596, Saudi Arabia; 2 Department of Community Health and Epidemiology, College of Medicine, University of Saskatchewan, Saskatoon, SK, S7N 5E5, Canada; E-Mail: Nazeem.Muhajarine@usask.ca

**Keywords:** attitude to health, anesthesia, early childhood caries, oral health, quality of life

## Abstract

*Aim:* To determine whether dental treatment under general anesthesia (GA) would improve quality of life for children as reported by Saudi Arabian parents using a Parental-Caregivers Perceptions Questionnaire (P-CPQ) and a Family Impact Scale (FIS). *Methods:* Sixty-six parents completed P-CPQ and FIS scales four to eight weeks after their children (ages three to ten years) underwent comprehensive dental treatment under GA. Postoperative data were compared with baseline data gathered before GA using paired *t*-test at the 0.05 level of significance. The responsiveness of the P-CPQ and the FIS and the magnitude of changes in children’s quality of life as a result of dental treatment were determined by calculating the effect size (ES). Cross-sectional construct validity and internal consistency were also examined using the pretreatment scores of the P-CPQ and the FIS scores. *Results:* The overall P-CPQ and FIS scores showed a significant decrease following treatment, concomitant with large ES in both scales and all their subscales with the exception of social wellbeing, which showed moderate ES (ES 0.59). The greatest relative changes were seen in the oral symptoms (ES 1.81) and the family activity (ES 1.57) subscales. *Conclusion:* Dental treatment under GA is associated with considerable improvement in children’s quality of life as perceived by Saudi parents. The P-CPQ and the FIS scales are valid and responsive to changes resulting from dental treatment of young children affected by severe childhood caries.

## 1. Introduction

Dental caries is the most common chronic disease in children. It is five to eight times more common than the second most common chronic disease in children—asthma. Despite the remarkable decrease in caries occurrence over the last few decades—particularly in developed countries—its high prevalence in developing countries and certain segments of the population (e.g., children and adolescents, the poor and near-poor, and underserved populations), burden (e.g., cost and absenteeism) and other ramifications (e.g., pain, lack of sleep, and, in rare cases, death) require more efforts from the public, organized dentistry, and governments to control the epidemic nature of oral disease [1,2].

Early childhood caries (ECC) is an aggressive form of caries affecting children under age six. The disease usually affects anterior teeth, rotting them to the gum line within a year, and if not treated will transmit to the posterior primary teeth, even affecting permanent teeth as they start to erupt at age six. Evidence shows that ECC, particularly in severe forms, adversely affects the quality of life in young children; caries can lead to pain, infection, abscesses, malnutrition, and gastrointestinal problems [3]. The most alarming manifestation of severe caries, which is often neglected, is how the children deal with their dental disease and how it affects their daily routines, including school performance, their ability to thrive, and their sense of self-esteem.

Despite the increased risk of treatment of ECC under general anesthesia (GA) and the wait times for operating rooms, which means delays in access to dental treatment for children in urgent need of dental care, GA is considered the most appropriate method for performing a complete rehabilitation of the child’s dentition in a single visit. Several studies evaluated the perceived outcomes and parental satisfaction following dental rehabilitation under GA [3,4]. Using a single page survey with simple dichotomous items, Acs *et al.* found that children with ECC receiving comprehensive dental treatment under GA achieved improvements in their quality of life and overall health [4]. In addition, a hierarchy of improvement was noted; the greatest being a reduction in pain experienced followed by improved quality in eating and in sleep. Parents were overwhelmingly satisfied with outcomes in their children as well in the process of care, reporting that their expectations had been met.

Given challenges associated with interacting with pediatric patients (e.g., children do not self-regulate their behavior nor are generally the initiators of health promotion and health care), it is not surprising that research on children’s health-related quality of life (HRQoL) has a short history. It can be traced back to 1990 when the Child Health Questionnaire (CHQ) was published. Canadian-French, German, and UK translations/adaptions of CHQ was then developed, evaluated, and validated in 1998 [5]. Conversely, children’s oral HRQoL (OHRQoL) is only now starting to develop, despite the significance of oral diseases in children’s lives. A number of OHRQoL scales have been developed and validated [6]. Because young children, in particular, are often not reliable source of medical information, parents are relied on as informants for measuring child OHRQoL [6]. This instrument consists of two tools: a Parental-Caregivers Perceptions Questionnaire (P-CPQ) and a Family Impact Scale (FIS). From one of the few studies conducted using P-CPQ and FIS, Gaynor and Thomson found that (1) the dental treatment of young children under GA is associated with considerable improvement in their OHRQoL; and (2) the P-CPQ and the FIS are valid and responsive to treatment-associated changes in young children with ECC [6]. The authors urge replication in international settings and propose further research would assist in deciding whether the P-CPQ and FIS are valid and responsive to treatment-associated changes in ethnically diverse samples and whether the concept of OHRQoL transcends cultural differences. The purpose of this study was, therefore, two-fold: (1) to assess whether oral rehabilitation of young Saudi children under GA improved OHRQoL for children and their families; and (2) to identify epidemiological factors that are associated with parental ratings of child’s OHRQoL and changes associated with dental treatment.

## 2. Materials and Methods

The study used a single group pre-and-post design to evaluate changes in OHRQoL following comprehensive dental treatment under GA. Recruited from a pediatric dental clinic in Riyadh, Saudi Arabia, participants were children ages 3–10 years with multiple carious lesions that required treatment under GA. The study was ethically approved by the Research Center of Riyadh Colleges of Dentistry and Pharmacy (FRP/2012/7) and registered by Australian New Zealand Clinical Trials Registry (ACTRN12613000255785). The children were consecutively recruited during their first oral health screening, indicating they required dental treatment under GA for reasons other than disabilities (e.g., very young, parent’s wish to complete dental treatment in a single session, extensive dental caries, history of negative behavior during dental treatment). Patients who had medical problems affecting daily activities were excluded. After detailing treatments to be provided, parents’ informed consents were obtained. The WHO standard for surveying caries that is based on dmft/DMFT (decayed, missing, or filled primary and permanent teeth) was followed [7]. Further, children were classified according to need for dental care: children who were in urgent treatment need (children who are in obvious pain at the time of examination, visible infection, gross swelling, or presence of pus), and those who had visible decay but without urgent treatment needs. The OHRQoL questionnaire, including the P-CPQ and FIS, was completed by either parent. Follow-up data were collected by the same parent at the recall appointment after 4 to 8 weeks of treatment completion. Data about parent respondent’s age and his/her level of education were also recorded.

The OHRQoL instrument used here consists of four sections. Section 1 consists of two items, asking about the oral health status in general (item 1) and how much the child’s overall wellbeing is affected by the condition of his oral health (item 2). Section 2 consists of 14 items, focusing on the signs and symptoms of oral disease the child experienced during the last three months. Section 3 consists of 19 items, on the effects of oral disease on the child’s feelings and everyday activities. Section 2 and Section 3 comprise a Parental-Caregivers Perceptions Questionnaire (P-CPQ—more on P-CPQ below). Section 4 consists of 14 items, focusing on the effects of child’s oral disease on parents and other family members; this is known as a Family Impact Scale (FIS—more on FIS below). All items are scored using 5-point Likert-type response options (0, Never; 1, Once or Twice; 2, Sometimes; 3, Often; 4, Every day or almost every day; 0, Don’t know). The P-CPQ consists of 33 items, measuring certain domains (or subscales): oral symptoms (OS, six items), functional limitations (FL, eight items), emotional wellbeing (EWB, nine items), and social wellbeing (SWB, ten items). The FIS scale consists of 14 items, measuring certain domains: family/parental activity (FA, five items), parental emotions (PE, four items), and family/ financial conflict (FC, five items). Following the back-translation method, the questionnaire was previously validated in a Saudi Arabian sample of parents. An Arabic version was developed, making it culturally relevant for the target population, and used [8].

To determine the sample size the lowest effect size (ES 0.28) reported by Gaynor and Thomson on family conflict was used [6]. With 80% power and 0.05 alpha, it was determined that 67 cases would be needed.

Change scores for the overall OHRQoL, P-CPQ, and FIS were computed by subtracting post treatment scores from pretreatment scores. Change scores were also calculated for each domain or subscale. Paired *t*-tests were used at *p* < 0.05 to test the statistical significance of the changes. The responsiveness of the questionnaire and the magnitude of changes were determined, based on Cohen [9], by dividing the mean of the change scores by the standard deviation of the pretreatment scores [10]. This calculation would give a dimensionless measure of the effect (effect size [ES] statistics of less than 0.2 indicate a small clinically meaningful magnitude of change, 0.2–0.7 a moderate change, and more than 0.7 a large change) [9]. As in the previously-reported development and application of the P-CPQ and FIS instruments [6,10,11], cross-sectional construct validity was examined by evaluating the association between the means of pretreatment scores and the rating for the question, *How much is your child’s overall wellbeing affected by the condition of his/her teeth, lips, jaws or mouth?* The Kruskal-Wallis test was used to test the statistical significance of the observed association. Internal consistency was also assessed using Cronbach’s alpha. The effect of epidemiological variables on pretreatment scores was assessed using multiple linear regression that had P-CPQ and FIS as dependent variables. The effects of parent respondent’s gender and his/her level of education on P-CPQ and FIS change score were analyzed using interaction analysis. All tests were run using IBM SPSS Statistics version 20 (IBM Corporation, Somers, NY, USA).

## 3. Results

Based on the inclusion criteria, seventy parent-child dyads attending a pediatric dentistry clinic were approached for potential recruitment into the study. OHRQoL questionnaires were completed at baseline by all parents (response rate 100%). Four parents were lost to follow up due to family moving to another area (retention rate 94.2%), and therefore 66 patients were retained and included in the analysis. A total of 25 fathers and 41 mothers (mean age 40.7, SD 8.72) completed both pretreatment and post treatment questionnaires. There were 66 child patients: 36 males and 30 females. The mean age was 6.13 years (SD 2.06) with a range from three to ten years. The mean dmft was 9.76 (SD 3.07; range 2 to 19) and the mean DMFT for 24 children who have permanent teeth was 3.25 (SD 2.04; range 0 to 7). Fifty-six children were classified as in need of urgent dental care and 10 as having visible decays but without urgent dental care needs. Table 1 summarizes the number of children examined and received the dental care and their pertinent data. Fillings were provided to all patients (mean 6.84, SD 2.88, range 1–15); pulp therapy (pulpotomy/pulpectomy) was provided to 54 patients (mean 2.75, SD 2.06, range 0–8); stainless steel crowns (SSCs) were provided to 24 patients (mean 0.81, SD 1.35, range 0–6). Noteworthy, all fillings were tooth-colored (composite resin or resin-modified glass ionomer); amalgam restorations were not used. Forty-one patients had one or more of their teeth being extracted (mean 2.01, SD 2.19, range 0–8).

**Table 1:** Characteristics of patients who received dental care under general anesthesia.

Number	Sex		Age (SD)	dmft (SD)		DMFT (SD)		Urgent Need		Insurance	
TOTAL	M	F						yes	no	yes	no
66	36	30	6.13 (2.06)	9.76 (3.07)		3.25 (2.04)		56	10	60	6

Notes: M: male; F: female; SD: standard deviation; dmft: Decayed, Missing, or Filled Primary Teeth; DMFT: Decayed, Missing, or Filled Permanent Teeth.

The two general questions asked how parents would rate the health of their child’s teeth, lips, jaws and mouth and how much parents believed a child’s overall wellbeing was affected by the condition of his/her teeth, lips, jaws or mouth. About half of the parents rated their children’s oral health at baseline as poor, with 30.3% and 16.7% as fair and good, respectively. About one third of the parents indicated they believe that there is “very much” a relationship between overall wellbeing and the condition of the child’s oral health. Interestingly, 7.6% believe there is no relationship between the two; 15.2%, 16.7%, and 31.8% think the relationship is “very little”, “some”, or “a lot”, respectively. The global transition item postoperatively asked parents to rate the child’s overall quality of life since the operation to fix his/her teeth. The distribution of responses to this item clustered around “improved a lot” (n = 61% or 92.4%). Two (3%) parents reported “improved a little” and three (4.5%) reported “no change.”

Data on construct validity and internal consistency are presented in Table 2. It is evident that mean overall P-CPQ and FIS scores as well as P-CPQ subscales were gradually increasing with parents’ positive responses to the global item, *How much is the child’s overall wellbeing affected by his/her mouth?* (responses ranging from “Not at all”, to “Very little”, to “Some”, to “A lot/Very much”). The gradients in scores were statistically significant for all scales and subscales, demonstrating monotonic positive correlation with higher response scores for the single wellbeing item and scores of the P-CPQ and FIS.

**Table 2:** Pretreatment Parental-Caregivers Perceptions Questionnaire (P-CPQ) scale (and subscale) and Family Impact Scale (FIS) and Cronbach’s alpha.

OHRQoL	*How much is your child’s overall wellbeing affected by the condition of his/her teeth, lips, jaws or mouth?*					
	Not at all	Very little	Some	A lot/Very much	*p*-value	Cronbach’s alpha
Number (%)	5 (7.6%)	10 (15.2%)	11 (16.7%)	40 (60.6%)	_	_
P-CPQ	4.4 (4.39)	27.6 (16.94)	35.42 (18.66)	40.34 (10.85)	0.001*	0.89
P-CPQ domain						
OS	1.4 (0.54)	8.1 (4.78)	10.5 (2.46)	11 (4.5)	0.001*	0.66
FL	1.6 (1.81)	7.7 (6.05)	10.8 (8.2)	13.0 (3.34)	0.001*	0.82
EWB	1.2 (2.16)	7.3 (6.6)	10.2 (4.54)	11.25 (7.0)	0.009*	0.82
SWB	0.20 (0.44)	4.5 (3.17)	3.92 (5.47)	5.09 (4.7)	0.02*	0.75
FIS	2.4 (2.7)	12.6 (8.27)	16.9 (8.46)	17.2 (7.79)	0.002*	0.81
P-CPQ + FIS	6.8 (4.43)	40.2 (24.12)	52.32 (23.51)	57.54 (15.48)	0.001*	0.91

Notes: OHRQoL: oral health-related quality of life; P-CPQ: parental-caregivers perceptions questionnaire; OS: oral symptoms; FL: functional limitations; EWB: emotional wellbeing; SWB: social wellbeing; FIS: family impact scale; FA: family activity; PE: parental emotions; FC: family conflict; *: Statistically significant, Scores are presented as mean (standard deviation).

Table 3 demonstrates the effect of some epidemiological factors on parents’ perceptions of children’s OHRQoL using an ordinary least squares linear regression, with pretreatment P-CPQ and FIS scores as separate dependent continuous variables. The epidemiological variables tested were child’s age and gender; parent respondent’s age, gender, and level of education; dmft; insurance status; and treatment urgency. From all factors examined, only age of the child had a significant negative influence on P-CPQ score (Standardized *B* = −0.40, *t* = −2.98, *p* = 0.004). Worse P-CPQ scores were observed among older children.

**Table 3:** Multiple linear regression of epidemiological variables effects on pretreatment P-CPQ and FIS scores.

Model		Unstandradized Coefficients		Standardized Coefficients	*t*	Sig
		B	Std Error	Beta		
P-CPQ^a^	(Constant)	36.31	25.81		1.40	0.16
	Patient’s sex	−2.63	4.34	−0.073	−0.60	0.54
	Patient’s age	−3.50	1.17	−0.40	−2.98	0.004*
	Respondent’s age	−0.21	0.28	−0.09	−0.75	0.45
	Respondent’s sex	−5.17	4.65	−0.14	−1.11	0.27
	Respondent’s education	−8.44	7.10	−0.14	−1.18	0.24
	Respondent’s age	−0.21	0.28	−0.09	−0.75	0.45
	dmft	−0.27	0.75	−0.04	−0.36	0.71
	Insurance	−1.00	7.40	−0.01	−0.13	0.89
	Urgency	−3.81	6.19	−0.07	−0.61	0.54
FIS^b^	(Constant)	8.92	8.05		1.10	0.27
	Patient’s sex	−2.13	1.35	−0.19	−1.57	0.12
	Patient’s age	−0.21	0.36	−0.08	−0.57	0.56
	Respondent’s age	−0.14	0.08	−0.22	−1.64	0.10
	Respondent’s sex	−1.61	1.45	−0.14	−1.11	0.27
	Respondent’s education	−1.10	2.21	−0.06	−0.49	0.62
	Respondent’s age	−0.14	0.08	−0.22	−1.64	0.10
	dmft	0.006	0.23	0.003	0.025	0.98
	Insurance	−0.65	2.30	−0.03	−0.28	0.77
	Urgency	−1.57	1.93	−0.10	−0.81	0.41

Notes: ^a^: Dependent variable: P-CPQ; ^b^: Dependent variable: FIS; *: Association significant at *p* < 0.05.

The mean P-CPQ scores, FIS scores, and their domains at baseline and follow-up with effect sizes are presented in Table 4. Significant declines were observed in P-CPQ and FIS scores, indicating positive effect of treatment, for all domains. The associated effect sizes for P-CPQ and FIS change scores reflected large changes (ranging from 1.81 to 0.59). Social wellbeing of P-CPQ alone demonstrated a moderate change (ES 0.59). The largest change score was observed for the oral symptoms subscale of the P-CPQ (ES 0.1.81) and the family activity subscale of the FIS (ES 1.57).

**Figure 1:**
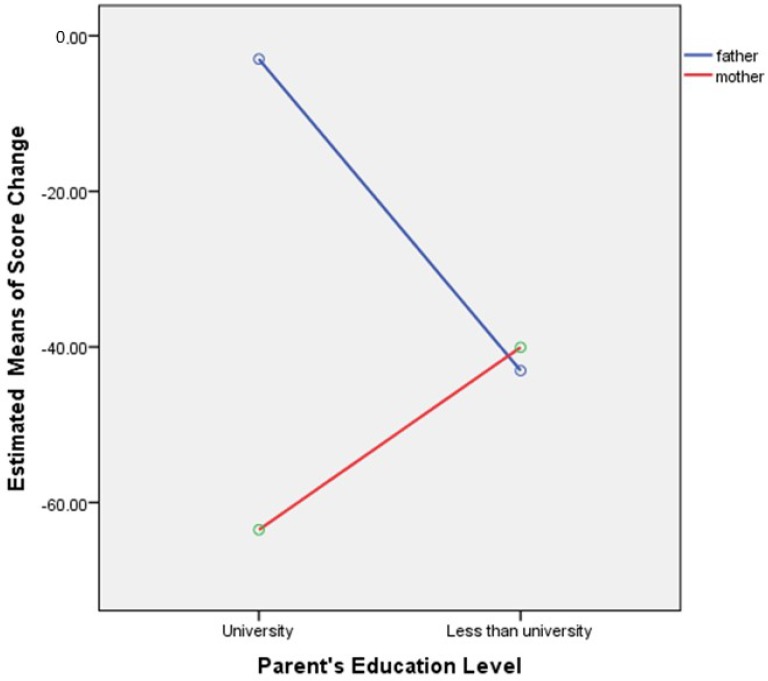
Effects of parent respondent’s sex and education on change score (Parental-Caregivers Perceptions Questionnaire (P-CPQ) plus Family Impact Scale (FIS)). Higher levels of education in mothers yielded higher estimated perceived positive effects as a result of dental treatment.

Figure 1 presents the effects of parent respondent’s gender and his/her level of education on score change. There was a significant interaction between respondents’ gender and level of education (*F*(1,65) = 11.83, *p* = 0.001). For mothers, higher level of education led to higher score change than lower level of education (*F*(1,65) = 12.08, *p* = 0.001). For fathers, level of education had no significant effect on change score (*F*(1,65) = 0.23, *p* = 0.62).

**Table 4:** Mean overall and domain scores in the Parental-Caregivers Perceptions Questionnaire (P-CPQ) and Family Impact Scale (FIS) at baseline and follow-up, change score, and effect sizes.

OHRQoL	OHRQoL mean scores (pretreatment/post treatment) and effect size					
	Pretreatment (SD)	Post treatment (SD)	Change in score (SD)	*p* value	Effect size (ES)	ES description
P-CPQ	33.37 (18.72)	4.42 (6.62)	28.95 (18.01)	<0.001*	1.54	Large
OS	9.69 (4.79)	1.0 (1.39)	8.69 (4.69)	<0.001*	1.81	Large
FL	10.03 (7.45)	1.72 (2.71)	8.30 (6.87)	<0.001*	1.11	Large
EWB	9.72 (6.86)	0.69 (1.86)	9.03 (6.99)	<0.001*	1.31	Large
SWB	3.92 (4.92)	1.0 (2.52)	2.92 (4.83)	<0.001*	0.59	Moderate
FIS	15.20 (8.85)	3.37 (3.81)	11.83 (9.03)	<0.001*	1.33	Large
FA	7.51 (4.1)	1.06 (1.67)	6.45 (4.31)	<0.001*	1.57	Large
PE	3.53 (2.66)	1.56 (1.91)	1.96 (2.79)	<0.001*	0.73	Large
FC	4.16 (3.90)	0.75 (1.62)	3.42 (3.93)	<0.001*	0.87	Large

Notes: OHRQoL: oral health-related quality of life; P-CPQ: parental-caregivers perceptions questionnaire; OS: oral symptoms; FL: functional limitations; EWB: emotional wellbeing; SWB: social wellbeing; FIS: family impact scale; FA: family activity; PE: parental emotions; FC: family conflict; SD: standard deviation; Change in score was calculated by subtracting post treatment scores from pretreatment scores; *: Statistically significant.

## 4. Discussion

OHRQoL was originally conceptualized in 1978 and is defined as that part of a person’s quality of life affected by the oral health [12]. This concept focuses on the patient as a whole and, therefore, emphasizes the holistic model of oral health. OHRQoL is assessed by either asking patients questions regarding person’s functioning (e.g., biting, chewing), sensation of pain, psychological (self-esteem), and social wellbeing, or having caregivers answer these questions for pediatric patients (proxy measurement). The latter approach was followed in the present study. Research demonstrated that parents’ assessment of their child’s OHRQoL correlated significantly with objective oral health indicators, such as dmf scores, as well as with their children’s self-ratings of OHRQoL [13]. Interestingly, one study that compared differences in perceptions of early childhood OHRQoL between fathers and mothers in Saudi Arabia found mothers were more suitable as proxy assessors than fathers in assessing the OHRQoL of their children (age range: 2.1 to 5.7 years) [8]. Comparing the mean number of “don’t know” responses between parents showed that fathers had less accurate knowledge of the OHRQoL of their children than did mothers [8]. In the present study 41 (62.1%) mothers and 25 (37.9%) fathers completed the questionnaires and there were no significant differences in scores between fathers and mothers.

Several cross-sectional studies investigated the impact of sociodemographic or socioenvironmental characteristics (e.g., sex, monthly family income, mother’s education, family structure, *etc.*) on OHRQoL in children [14,15]. Results have shown that these characteristics had different impacts on OHRQoL and, therefore, should be considered when planning strategies for children’s oral health. The current study found only age of the child was significantly associated with pretreatment P-CPQ scores where worse P-CPQ scores were observed among older children (6–10 years). A similar approach was recently followed by Pani *et al*. to explore the effects of different sociodemographic variables on parents’ perceptions of their children’s quality of life [12]. Regression analysis revealed that the significant factor influencing P-CPQ was maternal age, indicating that older mothers had greater concerns about children’s oral health. Maternal education played a role in FIS, while similar variables for the fathers did not. An inverse relationship was observed between the FIS and family finances [15]. Because child age was not included as a sociodemographic factor in Pani *et al*.’s study [15] and family income was not included in our study, direct comparison cannot be carried out. Another important difference between the two studies is that the study by Pani *et al*. included a sample of Saudi children who were autistic [15]. The current study included normal children who were otherwise affected by severe dental caries. Dental caries, resultant cavities and toothache/infection tend to increase as children grow up [2], and this may explain the significant association between child’s age and P-CPQ.

This study found that maternal education played a significant role in determining the positive effect of dental treatment on children’s OHRQoL, with higher level of education led to higher score change hence perception of better quality of life as a result of dental treatment. To the best of our knowledge, there are no published studies that evaluate the effect of sociodemographic variables on OHRQoL in interventional, longitudinal studies. This area of research warrants further investigation.

Many OHRQoL measures have been used in cross-sectional study designs; however, only a few have been used to assess change in OHRQoL using longitudinal study designs such as clinical trials. In principle, the longitudinal studies provide better evidence about causes of disease and effects of treatment than do cross-sectional studies by establishing a temporal sequence of events between a putative cause and subsequent effect [16]. The methods used here to measure change in health status were by computing change scores by subtracting pre-event (*i.e*., treatment) scores from post-event scores, and by making retrospective judgments about global change in OHRQoL by asking participants at some defined period after the event (four to eight weeks) to rate whether children’s OHRQoL has improved, stayed the same, or worsened. These methods, as discussed by Slade [16] based on Locker’s work [17], remove the statistical uncertainties about consistency in reference point by placing the onus on the respondents to judge the current OHRQoL and the earlier OHRQoL. It also helps characterize within-subject change. Because the vast majority of respondents in our study judged changes brought about by dental treatment as “much/a lot improvement,” the associations between retrospective judgments and computed change scores cannot be calculated, giving support to Locker’s statement [17], “Global transition judgments are simple to use but may not detect small to moderate changes.” Because of the lack of variation in responses to the post treatment global transition item: *Since the operation to fix his/her teeth, is your child’s overall quality of life: Much improved, A little improved, The same, A little worse, or Much worse*, assessment of longitudinal construct validity and calculation of the minimal important difference (MID) were hampered [11]. Coinciding with considerable change scores and large ES, the vast majority of parents (92.4%) reported considerable improvement as a result of dental treatment, supporting the validity of the instruments as an indicator of change.

Table 5 compares effect sizes reported in three studies [6,18,19], which used the same protocol and questionnaire adopted here, to the current study. It is evident that comprehensive dental treatment has a moderate to large positive effect on OHRQoL of children as perceived by their parents.

**Table 5:** Comparison of effect sizes from three previous studies to the current study.

OHRQoL	Gaynor & Thomson [6]	Malden *et al.* [18]	Jabarifar *et al.* [19]	Current study
P-CPQ	0.88	0.9	1.84	1.54
OS	1.22	1.3	2.98	1.81
FL	0.68	0.66	2.67	1.11
EWB	0.71	0.8	0.63	1.31
SWB	0.35	0.4	1.05	0.59
FIS	0.52	0.8	1.35	1.33
Age range	3–9	2.5–15.1	3–10	3–10

Notes: OHRQoL: oral health-related quality of life; P-CPQ: parental-caregivers perceptions questionnaire; OS: oral symptoms; FL: functional limitations; EWB: emotional wellbeing; SWB: social wellbeing; FIS: family impact scale.

Although clinically not significant, the differences in effect sizes among the four studies may reflect the effects of race and ethnicity on OHRQoL [20]. The two studies on two different samples of children from New Zealand reported comparable ES [6,18], while the Iranian study reported the highest ES [19]. The current study on Saudi children demonstrated ES somewhat in-between. The findings also corroborate our previous findings from the same Saudi sample but used short-form versions of the OHRQoL instruments [21]. It should be remembered, however, that children in these studies are among those with the worst dental conditions in their age group, as documented by the high dmf/DMF scores and the need for general anesthesia to complete the dental treatment. This means that the sensitivity and responsiveness of the OHRQoL instrument to more subtle differences and changes in child oral health warrants further investigation. A point worth mentioning is the short-term evaluation; longer time follow-up is required to ascertain the long-term improvement in children’s quality of life as a result of dental treatment. Understanding the effect of quality of life (QoL) will require assessment of health over time. We opted in this study to complete the post treatment questionnaires four to eight weeks after treatment, enabling comparisons with studies, which followed a similar protocol. In addition, it was thought that within the first few postoperative days some patients could experience a deterioration in their quality of life that will not extend beyond the traditionally recognized side effects, which will show significant improvement in the first postoperative week. While it is true that, by maintaining good oral health, patients and families can enjoy positive effects associated with the dental treatment, data collected after longer period of follow-up would ascertain long-term effects of dental treatment on OHRQoL (this is a work in progress). Peterson correctly stated that, after all the QoL action is between the individual, and his ownership of oral health and compliance with home care instructions, and what the real world affords—external attributions considered as structural factors that also determine QoL and wellbeing [22]. Finally, a limitation to the current study is the lack of a comparison group, which did not receive treatment. This would have allowed comparison of changes of scores as time passes by.

A 2012 study assessed the impact of dental treatment of severe dental caries on Saudi children’s weight, height and subjective health related outcomes, including dental pain, satisfaction with teeth and smile, dental sepsis and child’s appetite compared to control schoolchildren with untreated dental caries [23]. There were insignificant improvements in anthropometric outcomes between the groups after treatment of caries. However, treated children had significantly less pain and higher satisfaction compared to controls. Controls had significantly poorer appetites compared to treated children. All treated children were free of clinical dental sepsis, whereas 20% of controls who were free of sepsis at baseline had sepsis at follow-up [23]. Research has shown that teeth with infected pulps negatively affect children’s eating and sleeping [23]. Pulpally-involved teeth are also linked to a higher level of inflammation, which in turn contributes to undermining immunity, leading to anemia and growth failure [24]. Some research has found that extracting of severely decayed teeth in underweight Filipino children was associated with weight gain after 4 month. In addition, decreases in oral health impacts on sleeping after tooth extraction appeared to be most strongly associated with weight gain [25]. Our study included children with severe dental caries. Children below age 6 were affected by severe ECC, whereas older children had severe dental caries by having at least 2 teeth with pulpal involvement, as defined by Alkarimi *et al.* [23]. Severe dental caries is not uncommon in Saudi Arabian children [2]. Preventive measures should be improved to reduce necessity of invasive procedures such as dental treatment under GA.

Many parents may express some level of anxiety or concern before and during GA, having heard about serious morbidity or even rare cases of mortality during GA. Research indicates that postoperative morbidity in pediatric patients under GA for dental rehabilitation in Saudi Arabia was common, but mostly of mild severity and limited to the first day [26]. A significant reduction in complaints was reported by parents by the third postoperative day and, as shown by the current study, dental treatment under GA can thereafter have significant positive effects on the quality of life for children and their families.

Answering the questions on child’s SWB, some parents expressed some objections to the appearance of SSCs although the rationale for using them had been fully explained to children and parents during treatment plan. Bell *et al*. reported that children treated with SSCs rated them as ‘being different’ while 5% of parents expressed strong objections to the appearance [27]. Aesthetic alternatives to conventional SSCs, such as pre-veneered SSCs or adhesive restorations, should be considered to address this issue [28].

Dental treatment under GA is not covered by all medical insurance companies in Saudi Arabia, and there is a long list of pediatric dental patients waiting to undergo GA in Ministry of Health and Ministry of Higher Education hospitals, which provide such treatment free of charge. Dental care is the most prevalent unmet healthcare need for children in Saudi Arabia, as is the case in many other countries [29]. Research on children’s OHRQoL can help demonstrate the burden of illness caused by oral diseases, which might be useful for changing health policy. Treatment outcomes should be evaluated based on QoL, morbidity, mortality and cost considerations. Appraisals of HRQoL are generally considered to include both positive and negative aspects of an experience that may have differential effects on overall ratings over time. Longitudinal assessments of HRQoL provide greater understanding regarding how HRQoL changes as an individual proceeds through stages of health, illness, treatment, and recovery. There currently is heightened emphasis on OHRQoL outcomes; research in this area is still in its formative stages. The influence of culture on perceptions of HRQoL and the use of quantitative and qualitative approaches to measurement must be further analyzed to ensure more comprehensive and valid assessments.

## 5. Conclusion

The dental treatment of children under GA is associated with significant improvement of OHRQoL as perceived by Saudi Arabian children’s parents. Both the P-CPQ and the FIS are valid and responsive to changes brought about by dental treatment of young children affected by severe childhood caries.
